# Supplementary material for: Female genital mutilation and safer sex negotiation among women in sexual unions in sub-Saharan Africa: Analysis of demographic and health survey data
Source: PLoS One. 2024 May 17;19(5):e0299034. doi: 10.1371/journal.pone.0299034 (PMC11101093; doi:10.1371/journal.pone.0299034)
Supplement: S1 File — (DOCX) [file pone.0299034.s001.docx]

Supplementary file

**Table S1: Mixed effect analysis female genital cutting women’s ability to refuse partner’s sex in sub-Saharan Africa**

| **Variables** | **Model O** | **Model I**  **aOR [95% CI]** | **Model II**  **aOR [95% CI]** | **Model III**  **aOR [95% CI]** |
| --- | --- | --- | --- | --- |
| **Fixed effect results** |  |  |  |  |
| **FGM** |  |  |  |  |
| Not undergone FGM |  | 1.00 |  | 1.00 |
| Undergone FGM |  | 1.06^*^ [1.01,1.10] |  | 0.91^***^ [0.86,0.96] |
| **Maternal age (years)** |  |  |  |  |
| 15-19 |  | 1.00 |  | 1.00 |
| 20-24 |  | 1.08 [0.98,1.19] |  | 1.04 [0.95,1.15] |
| 25-29 |  | 1.11^*^ [1.01,1.23] |  | 1.04 [0.94,1.15] |
| 30-34 |  | 1.15^**^ [1.04,1.28] |  | 1.05 [0.94,1.17] |
| 35-39 |  | 1.26^***^ [1.13,1.40] |  | 1.09 [0.98,1.22] |
| 40-44 |  | 1.29^***^ [1.15,1.45] |  | 1.12 [0.99,1.26] |
| 45-49 |  | 1.24^***^ [1.10,1.40] |  | 1.05 [0.92,1.19] |
| **Marital status** |  |  |  |  |
| Married |  | 1.00 |  | 1.00 |
| Cohabiting |  | 1.87^***^ [1.70,2.05] |  | 1.33^***^ [1.20,1.46] |
| **Maternal educational level** |  |  |  |  |
| No education |  | 1.00 |  | 1.00 |
| Primary |  | 1.26^***^ [1.19,1.33] |  | 1.24^***^ [1.17,1.31] |
| Secondary |  | 1.67^***^ [1.56,1.79] |  | 1.47^***^ [1.37,1.59] |
| Higher |  | 2.39^***^ [2.10,2.73] |  | 2.03^***^ [1.78,2.33] |
| **Current working status** |  |  |  |  |
| No |  | 1.00 |  | 1.00 |
| Yes |  | 1.29^***^ [1.24,1.35] |  | 1.06^*^ [1.01,1.11] |
| **Religion** |  |  |  |  |
| Christianity |  | 1.00 |  | 1.00 |
| Islamic |  | 0.45^***^ [0.43,0.48] |  | 0.55^***^ [0.52,0.59] |
| African Traditional |  | 0.85^*^ [0.75,0.97] |  | 0.93 [0.82,1.07] |
| No religion |  | 0.63^***^ [0.52,0.78] |  | 0.64^***^ [0.52,0.79] |
| Others |  | 1.38 [0.67,2.85] |  | 1.52 [0.73,3.16] |
| **Comprehensive HIV and AIDS knowledge** | |  |  |  |
| No |  | 1.00 |  | 1.00 |
| Yes |  | 1.29^***^ [1.23,1.34] |  | 1.18^***^ [1.14,1.24] |
| **Partner’s age (years)** |  |  |  |  |
| 15-24 |  | 1.00 |  | 1.00 |
| 25-34 |  | 0.94 [0.84,1.06] |  | 0.96 [0.85,1.09] |
| 35-44 |  | 0.87^*^ [0.77,0.99] |  | 0.91 [0.80,1.04] |
| 45+ |  | 0.77^***^ [0.68,0.88] |  | 0.85^*^ [0.74,0.97] |
| **Partner’s educational level** |  |  |  |  |
| No education |  | 1.00 |  | 1.00 |
| Primary |  | 1.21^***^ [1.14,1.28] |  | 1.14^***^ [1.07,1.22] |
| Secondary |  | 1.48^***^ [1.39,1.57] |  | 1.19^***^ [1.12,1.27] |
| Higher |  | 1.50^***^ [1.37,1.64] |  | 1.21^***^ [1.10,1.33] |
| **Frequency of watching television** | |  |  |  |
| None at all |  | 1.00 |  | 1.00 |
| Less than once a week |  | 0.92^**^ [0.86,0.97] |  | 1.06 [1.00,1.13] |
| At least once a week |  | 0.88^***^ [0.83,0.93] |  | 1.15^***^ [1.08,1.23] |
| **Frequency of listening to radio** | |  |  |  |
| None at all |  | 1.00 |  | 1.00 |
| Less than once a week |  | 1.04 [0.98,1.10] |  | 1.15^***^ [1.09,1.23] |
| At least once a week |  | 1.02 [0.97,1.07] |  | 1.20^***^ [1.14,1.26] |
| **Frequency of reading newspaper/magazine** | |  |  |  |
| None at all |  | 1.00 |  | 1.00 |
| Less than once a week |  | 1.08 [0.99,1.17] |  | 1.16^***^ [1.06,1.27] |
| At least once a week |  | 0.96 [0.86,1.07] |  | 1.10 [0.98,1.23] |
| **Wealth index** |  |  |  |  |
| Poorest |  |  | 1.00 | 1.00 |
| Poorer |  |  | 1.15^***^ [1.08,1.22] | 1.01 [0.95,1.07] |
| Middle |  |  | 1.23^***^ [1.16,1.31] | 0.99 [0.93,1.06] |
| Richer |  |  | 1.44^***^ [1.34,1.54] | 1.03 [0.96,1.11] |
| Richest |  |  | 1.91^***^ [1.77,2.07] | 1.06 [0.96,1.16] |
| **Residence** |  |  |  |  |
| Urban |  |  | 1.00 | 1.00 |
| Rural |  |  | 0.82^***^ [0.78,0.87] | 0.89^***^ [0.84,0.94] |
| **Countries** |  |  |  |  |
| Burkina Faso |  |  | 1.00 | 1.00 |
| Ethiopia |  |  | 0.932 [0.85,1.03] | 0.73^***^ [0.65,0.81] |
| Gambia |  |  | 0.92 [0.83,1.02] | 0.89^*^ [0.80,1.00] |
| Guinea |  |  | 0.81^***^ [0.74,0.88] | 0.93 [0.84,1.02] |
| Kenya |  |  | 2.46^***^ [2.27,2.65] | 1.13^*^ [1.03,1.25] |
| Liberia |  |  | 5.08^***^ [4.49,5.74] | 2.53^***^ [2.20,2.90] |
| Mali |  |  | 0.30^***^ [0.27,0.33] | 0.34^***^ [0.30,0.38] |
| Nigeria |  |  | 1.44^***^ [1.35,1.54] | 1.04 [0.96,1.13] |
| Sierra Leone |  |  | 2.24^***^ [2.08,2.41] | 2.21^***^ [2.04,2.40] |
| Senegal |  |  | 0.34^***^ [0.31,0.37] | 0.35^***^[0.32,0.38] |
| Togo |  |  | 2.37^***^ [2.14,2.62] | 1.44^***^ [1.28,1.61] |
| **Random effect results** |  |  |  |  |
| PSU variance (95% CI) | 0.41 [0.35, 0.47] | 0.17 [0.15, 0.21] | 0.27 [0.23, 0.31] | 0.20 [0.17, 0.23] |
| ICC | 0.11 | 0.05 | 0.08 | 0.06 |
| LR Test | 1242.69 (<0.001) | 614.37 (<0.001) | 933.46 (<0.001) | 694.55 (<0.001) |
| Wald chi-square | Reference | 4320.13*** | 4587.29*** | 5811.30*** |
| **Model fitness** |  |  |  |  |
| Log-likelihood | -33288.29 | -30859.26 | -30630.88 | -29813.45 |
| AIC | 66580.59 | 61780.51 | 61295.77 | 59718.91 |
| N | 50,337 | 50,337 | 50,337 | 50,337 |
| Number of clusters | 1,608 | 1,608 | 1,608 | 1,608 |

aOR= adjusted odds ratios; CI Confidence Interval; ^*^ *p* < 0.05, ^**^ *p* < 0.01, ^***^ *p* < 0.001; 1 = Reference category; PSU=Primary Sampling Unit; ICC = Intra-Class Correlation; LR Test= Likelihood ratio Test; AIC = Akaike’s Information Criterion

**Table S2: Mixed effect analysis female genital cutting women’s ability to ask partner to use condom in sub-Saharan Africa**

| **Variables** | **Model O** | **Model I**  **aOR [95% CI]** | **Model II**  **aOR [95% CI]** | **Model III**  **aOR [95% CI]** |
| --- | --- | --- | --- | --- |
| Fixed effect results |  |  |  |  |
| **FGM** |  |  |  |  |
| Not undergone FGM |  | 1.00 |  | 1.00 |
| Undergone FGM |  | 0.89^***^ [0.85,0.93] |  | 0.82^***^ [0.78,0.86] |
| **Maternal age (years)** |  |  |  |  |
| 15-19 |  | 1.00 |  | 1.00 |
| 20-24 |  | 1.12^*^ [1.02,1.23] |  | 1.05 [0.96,1.16] |
| 25-29 |  | 1.19^***^ [1.08,1.31] |  | 1.07 [0.97,1.18] |
| 30-34 |  | 1.21^***^ [1.09,1.34] |  | 1.07 [0.96,1.19] |
| 35-39 |  | 1.21^***^ [1.08,1.34] |  | 1.04 [0.93,1.16] |
| 40-44 |  | 1.19^**^ [1.06,1.34] |  | 1.04 [0.92,1.17] |
| 45-49 |  | 1.08 [0.95,1.22] |  | 0.94 [0.83,1.06] |
| **Marital status** |  |  |  |  |
| Married |  | 1.00 |  | 1.00 |
| Cohabiting |  | 1.41^***^ [1.30,1.53] |  | 1.40^***^ [1.28,1.53] |
| **Maternal educational level** |  |  |  |  |
| No education |  | 1.00 |  | 1.00 |
| Primary |  | 1.57^***^ [1.48,1.66] |  | 1.37^***^ [1.29,1.45] |
| Secondary |  | 1.82^***^ [1.71,1.95] |  | 1.59^***^ [1.49,1.71] |
| Higher |  | 2.22^***^ [1.96,2.51] |  | 2.03^***^ [1.78,2.31] |
| **Current working status** |  |  |  |  |
| No |  | 1.00 |  | 1.00 |
| Yes |  | 1.23^***^ [1.18,1.29] |  | 1.17^***^ [1.12,1.23] |
| **Religion** |  |  |  |  |
| Christianity |  | 1.00 |  | 1.00 |
| Islamic |  | 0.57^***^ [0.54,0.60] |  | 0.62^***^ [0.59,0.66] |
| African Traditional |  | 0.54^***^ [0.47,0.62] |  | 0.52^***^ [0.45,0.60] |
| No religion |  | 0.75^**^ [0.61,0.93] |  | 0.62^***^ [0.50,0.77] |
| Others |  | 2.26^*^ [1.07,4.81] |  | 2.28^*^ [1.07,4.87] |
| **Comprehensive HIV and AIDS knowledge** | |  |  |  |
| No |  | 1.00 |  | 1.00 |
| Yes |  | 1.35^***^ [1.30,1.41] |  | 1.30^***^ [1.24,1.35] |
| **Partner’s age (years)** |  |  |  |  |
| 15-24 |  | 1.00 |  | 1.00 |
| 25-34 |  | 0.90 [0.80,1.02] |  | 0.92 [0.82,1.04] |
| 35-44 |  | 0.79^***^ [0.70,0.89] |  | 0.84^* *^[0.74,0.95] |
| 45+ |  | 0.64^***^ [0.56,0.73] |  | 0.69^***^ [0.60,0.79] |
| **Partner’s educational level** |  |  |  |  |
| No education |  | 1.00 |  | 1.00 |
| Primary |  | 1.37^***^ [1.29,1.45] |  | 1.24^***^ [1.16,1.32] |
| Secondary |  | 1.56^***^ [1.47,1.66] |  | 1.40^***^ [1.32,1.49] |
| Higher |  | 1.56^***^ [1.43,1.70] |  | 1.43^***^ [1.31,1.57] |
| **Frequency of watching television** | |  |  |  |
| None at all |  | 1.00 |  | 1.00 |
| Less than once a week |  | 1.18^***^ [1.11,1.25] |  | 1.20^***^ [1.12,1.27] |
| At least once a week |  | 1.30^***^ [1.23,1.37] |  | 1.25^***^ [1.18,1.34] |
| **Frequency of listening to radio** | |  |  |  |
| None at all |  | 1.00 |  | 1.00 |
| Less than once a week |  | 1.05 [0.99,1.11] |  | 1.06 [1.00,1.12] |
| At least once a week |  | 1.19^***^ [1.14,1.25] |  | 1.15^***^ [1.09,1.21] |
| **Frequency of reading newspaper/magazine** | |  |  |  |
| None at all |  | 1.00 |  | 1.00 |
| Less than once a week |  | 1.29^***^ [1.18,1.40] |  | 1.29^***^ [1.18,1.40] |
| At least once a week |  | 1.35^***^ [1.21,1.50] |  | 1.32^***^ [1.18,1.47] |
| **Wealth index** |  |  |  |  |
| Poorest |  |  | 1.00 | 1.00 |
| Poorer |  |  | 1.29^***^ [1.21,1.37] | 1.07^*^ [1.00,1.14] |
| Middle |  |  | 1.54^***^ [1.45,1.64] | 1.13^***^ [1.06,1.21] |
| Richer |  |  | 2.03^***^ [1.89,2.17] | 1.25^***^ [1.16,1.35] |
| Richest |  |  | 3.14^***^ [2.91,3.40] | 1.38^***^ [1.26,1.51] |
| **Residence** |  |  |  |  |
| Urban |  |  | 1.00 | 1.00 |
| Rural |  |  | 0.79^***^ [0.75,0.83] | 0.88^***^ [0.83,0.93] |
| **Countries** |  |  |  |  |
| Burkina Faso |  |  | 1.00 | 1.00 |
| Ethiopia |  |  | 1.05 [0.95,1.16] | 0.73^***^ [0.65,0.81] |
| Gambia |  |  | 2.23^***^ [2.01,2.46] | 1.85^***^ [1.65,2.07] |
| Guinea |  |  | 0.68^***^ [0.62,0.75] | 0.71^***^ [0.64,0.79] |
| Kenya |  |  | 4.82^***^ [4.46,5.22] | 1.76^***^ [1.60,1.94] |
| Liberia |  |  | 2.52^***^ [2.28,2.77] | 0.99 [0.88,1.11] |
| Mali |  |  | 0.75^***^ [0.67,0.83] | 0.75^***^ [0.68,0.84] |
| Nigeria |  |  | 1.34^***^ [1.25,1.43] | 0.74^***^ [0.68,0.80] |
| Sierra Leone |  |  | 1.77^***^ [1.65,1.90] | 1.51^***^ [1.40,1.64] |
| Senegal |  |  | 0.82^***^ [0.76,0.88] | 0.70^***^ [0.64,0.77] |
| Togo |  |  | 3.05^***^ [2.77,3.36] | 1.67^***^ [1.49,1.87] |
| **Random effect results** |  |  |  |  |
| PSU variance (95% CI) | 0.60 [0.52, 0.68] | 0.22 [0.19, 0.26] | 0.26 [0.22, 0.30] | 0.22 [0.18, 0.26] |
| ICC | 0.15 | 0.06 | 0.07 | 0.06 |
| LR Test | 1862.62 (<0.001) | 775.82 (<0.001) | 897.69 (<0.001) | 735.93 (<0.001) |
| Wald chi-square | Reference | 5805.88*** | 4523.40*** | 6552.77*** |
| **Model fitness** |  |  |  |  |
| Log-likelihood | -33959.44 | -30671.34 | -31430.52 | -30050.18 |
| AIC | 67922.87 | 61404.67 | 62895.04 | 60192.35 |
| N | 50,337 | 50,337 | 50,337 | 50,337 |
| Number of clusters | 1,608 | 1,608 | 1,608 | 1,608 |

aOR= adjusted odds ratios; CI = Confidence Interval; ^*^ *p* < 0.05, ^**^ *p* < 0.01, ^***^ *p* < 0.001; 1 = Reference category; PSU=Primary Sampling Unit; ICC = Intra-Class Correlation; LR Test= Likelihood ratio Test; AIC = Akaike’s Information Criterion
