# Supplementary material for: Female genital mutilation and safer sex negotiation among women in sexual unions in sub-Saharan Africa: Analysis of demographic and health survey data
Source: PLoS One. 2024 May 17;19(5):e0299034. doi: 10.1371/journal.pone.0299034 (PMC11101093; doi:10.1371/journal.pone.0299034)
Supplement: Attachment — Submitted filename: Reviewers comments on FGM & safer sex negotiation (1107).docx [file pone.0299034.s002.docx]

COMMENTS

Reviewer #1: The authors have tackled a very important societal practice that has short and long term physical and psychological impact on women and girls. Their use of multi-country DHS dataset is also commendable as it presents an opportunity to understand how widespread the FGM practice is and also examine its impact in a diverse setting. However, the authors have not done a robust analysis of the dataset. Below are some concerns and suggestions.

Our response: Thank you. We have addressed all the comments raised.

The main concern is that the authors have not discussed the underlying mechanisms and linkages between FGM and other socio-demographic factors, considering that FGM is more likely to be done before marriage (<15yrs). The paper would benefit from some in-depth discussion of the link between FGM and the outcomes as well as link between FGM and socio-demographic factors. Two-thirds of the discussion is on the association between the outcomes and socio-demographic factors, with little focus on FGM (as their main exposure), which is the focus of the paper.

Our response: Thank you very much. The discussion section has been revised to provide more information on FGM and safer sex negotiation, FGM and socio-demographic factors, and safer sex negotiation and socio-demographic factors.

The other concern is on the analysis procedure. Much as it is important to look at the overall association between FGM and the outcomes, the analysis would have benefitted from individual country estimates, considering the wide variation in FGM (6.9% in Togo vs 96.3 % in Guinea) as well as safe sex practices across settings (30% in Mali vs 85% in Liberia). It is not clear if the authors examined the heterogeneity in the both the outcome and main exposure across the countries and how this was accounted for in the pooled analysis. It would important to examine how the risk of unsafe sex varies across the different settings.

Our response: Thank you. We have provided a new result, which examined the association between FGM and safer sex negotiation by country (Table 4).

In the data analysis section, the authors outlined four models they fitted but do not explain to the reader the criteria used in selecting the two models they present in table 3.

Our response: Thank you. We have attached the complete tables containing the four models as supplementary file. We presented the results for only the two models because, the last models were the best-fitted models based on the lowest value of the Akaike’s Information Criterion and the highest value of the log-likelihood. Please refer to the supplementary file for detailed information.
