# Supplementary material for: Female genital mutilation and safer sex negotiation among women in sexual unions in sub-Saharan Africa: Analysis of demographic and health survey data
Source: PLoS One. 2024 May 17;19(5):e0299034. doi: 10.1371/journal.pone.0299034 (PMC11101093; doi:10.1371/journal.pone.0299034)
Supplement: Attachment — Submitted filename: Reviewers comment.docx [file pone.0299034.s003.docx]

Reviewer #2: This article used data from more than 50,000 currently married and cohabiting women aged 15-49 in 11 countries in SSA from the Demographic and Health Surveys to investigate the relationship between female genital mutilation and safe sex negotiation. The findings are socially relevant given the continuing issue of female genital mutilation in the region. Overall, the paper is well-written and the conclusions are supported by the results presented. However, there are areas where the manuscript can be improved:

Response: Thank you.

1. Some slight errors can be found in the text. For instance, see line 80 and line 170.
Response: We have corrected these errors.

2. The improved discussion in the end is helpful. However, it would help the reader to highlight the theorized connection between female genital mutilation and safe sex negotiation in the introduction. Another option could be to have a small theoretical framework after the introduction, which highlights how the two are hypothesized to be related. Again, which mechanisms are at stake?

Response: Thank you for this observation. A theoretical perspective has been provided.

3. It is argued that the association between female genital mutilation and women’s ability to negotiate for safer sex in sub-Saharan Africa is investigated. However, the focus is just on 11 countries in sub-Saharan Africa. Including the Multiple Indicator Cluster Surveys would make the analysis more robust and accurate to make conclusions about the entire region. This would benefit the cross-validation of the findings. I understand that this could be beyond the scope of this analysis, but I could not refrain from wondering whether the results would hold when including more countries in sub-Saharan Africa. Can the authors explain why they did not use the MICS data and state how many countries were excluded as a result?

Response: Thank you. We understand that data on FGM is available in the MICS. However, the focus of this study is to use the standard DHS to ascertain the association between FGM and safe sex negotiation. Also, there were variations in the surveys years for the MICS and DHS, which we think could have affected the study. Hence the decision to use only the DHS. As at the time of this study, all the countries that had data on both FGM and safe sex negotiation as well as the covariates.

4. To the reader, various components of the analysis are unclear. For instance, how is the weighting of the data being taken into account in the analysis? So, what exact weighting procedure was used? If the DHS weights are used, one is given more weight to surveys with more data (nationally representative for each country, but not across countries). See the DHS sampling manual: https://dhsprogram.com/pubs/pdf/DHSM4/DHS6_Sampling_Manual_Sept2012_DHSM4.pdf . The suggested approach is to de-normalize and renormalize according to the relevant measure. Next to that, it is unclear whether multilevel analysis was performed. This is not mentioned in the paper, however, the results (intraclass correlation) suggest that this was performed. Finally, can information regarding multicollinearity be provided?

Response: We took into account weighting at the country and pooled levels. Thus, we de-normalised and renormalised the dataset before the final analysis. Multilevel regression analysis was used to examine the association between FGM and safer sex negotiation. We also checked for evidence of collinearity and there was no evidence of high collinearity among the variables. The minimum, maximum, and mean variance inflation factor were 1.00, 3.74, and 1.84, respectively.

5. Regarding Table 4, I am wondering why models 1 and 2 are being shown. Before, it was argued that model 3 was the best estimation. So, why is this not used to perform the country analysis? Also, look at PLOS ONE guidelines for statistical reporting, https://journals.plos.org/plosone/s/submission-guidelines.#loc-statistical-reporting. This suggests including the complete estimations with particular format requirements in the appendix.

Response: Thank you. We have attached the complete table indicating Model O, I, II, and III as a supplementary file.

6. In the discussion, it is argued that both female genital mutilation and safe sex negotiation are related to social norms. Could the authors take this into account in the statistical analysis?

Response: Thank you for your observation. This was beyond the scope of the study, hence, it has been captured as a limitation.

7. The results in ‘prevalence of female genital mutilation and safer sex negotiation among women in sub-Saharan Africa’ do not align with the results mentioned in the abstract.

Response: We have corrected this error.
