# Supplementary material for: Female genital mutilation and safer sex negotiation among women in sexual unions in sub-Saharan Africa: Analysis of demographic and health survey data
Source: PLoS One. 2024 May 17;19(5):e0299034. doi: 10.1371/journal.pone.0299034 (PMC11101093; doi:10.1371/journal.pone.0299034)
Supplement: Attachment — Submitted filename: Reviewers Comment_R2.docx [file pone.0299034.s004.docx]

**Response to Reviewers Comments**

Reviewer #2:

Thank you for the response to the comments that were made. The paper is well-written and conclusions are supported by the results presented. Many of the comments made were addressed appropriately! Some additional minor remarks:

- One more minor error in line 215

Response: We have corrected this error.

- Regarding the MICS surveys, I am not sure whether I understand the arguments made. So, why was the DHS chosen in particular? And what do you mean by variations in the survey years for the MICS? Anyhow, explaining why the MICS were not used and the possible downslides of this could be discussed in the limitation parts of the article.

Response: Thank you. We used the standard DHS because tot all the countries have the MICS dataset. Additionally, the data collection process for the MICS and the variable measurement except for the sociodemographic characteristics differs from the standard DHS. Hence, our decision to use the standard DHS dataset. However, we have acknowledged this as a limitation.

- Thank you for responding to several of the questions regarding to the analysis and adjustments made to the paper. However, the information about the weighting is still not included in the article itself. I think it would be good for transparency and potential reproducibility of the methods and results to add these to the article as well.

Response: Thank you. We have described how the weighting methodology used in the study.

- The statement in line 247 is only true for particular countries as well.

Response: We have corrected this by adding the countries.
